# Atherogenic Index of Plasma and Anthropometric Measurements among Osteoporotic Postmenopausal Sudanese Women: Possible Risk for Cardiovascular Disease

**DOI:** 10.1155/2022/1545127

**Published:** 2022-09-26

**Authors:** Abdelgadir Elmugadam, Ghada A. Elfadil, Abdalrahman Ismail Hamad, Ahlam Badreldin El Shikieri, Mawahib Aledrissy, Hisham N. Altayb

**Affiliations:** ^1^Department of Clinical Chemistry, College of Medical Laboratory Science, Sudan University of Science and Technology, Khartoum, Sudan; ^2^Clinical Nutrition Department, Taibah University, Medina, Saudi Arabia; ^3^Faculty of Medicine, National Ribat University, Khartoum, Sudan; ^4^Department of Biochemistry, Faculty of Science, King Abdulaziz University, Jeddah, Saudi Arabia

## Abstract

**Introduction:**

Data examining the health of menopausal women and the prevalence of osteoporosis remain to be limited in Africa, especially in sub-Saharan countries. Thus, in this current study, we aimed to assess the atherogenic index of plasma (AIP) and anthropometric measurements of osteoporotic postmenopausal women and determine their risk for cardiovascular disease (CVD).

**Methods:**

This is a cross-sectional, community-based study. Postmenopausal women (*n* = 300), aged ≥45 years, were recruited from Khartoum state, Sudan. Dual-energy X-ray absorptiometry was used to assess bone density. Weight, height, and waist circumference were measured twice. Fasting blood samples (5 ml) were collected to determine total cholesterol (TC), triglycerides (TG), low-density lipoprotein cholesterol (LDL-C), and high-density lipoprotein cholesterol (HDL-C). AIP was calculated as an indicator of CVD risk.

**Results:**

The mean age of the postmenopausal women was 61.6 ± 10.2 years (range 47–90 years). Women (*n* = 80) had the normal *T*-score, and an equal number had osteoporosis (*n* = 110) and osteopenia (*n* = 110). The prevalence of osteoporosis was 36.7%. Many postmenopausal women with normal *T*-scores suffered from general (71.2%) and central (94%) obesity. Postmenopausal women had high TC (24.4%), TG (25.6%), LDL-C (13.7%), and low HDL-C (76.0%) levels. Osteoporotic women (36.4%) were found to have a medium to high risk of CVD as determined by AIP. Women with normal *T*-scores had a higher number of CVD risk factors. A positive correlation was noted between AIP and TC among osteopenic (*r* = 0.292; *P*=0.002) and osteoporotic women (*r* = 0.265; *P* < 0.001).

**Conclusion:**

Osteoporosis was prevalent among Sudanese postmenopausal women who also had an increased risk for CVD. Public health education about osteoporosis and CVD risk is thus recommended.

## 1. Introduction

During menopause, which is defined as the end of a woman's reproductive life, ovaries fail to produce enough estrogen. Consequently, postmenopausal women are prone to diseases including osteoporosis (OP), cardiovascular disease (CVD), and dyslipidemia [1]. Osteoporosis, a bone-thinning illness, can lead to fractures; it has emerged as a major public health concern [2]. Approximately 200 million people suffer from OP worldwide, with 9 million osteoporotic fractures occurring per year, 1.6 million of which impact the hip joint [3]. Low bone mineral density (BMD) has been identified as the primary cause of OP [4]. Aging, being female, having reduced physical activity levels, low calcium intake, and hypothyroidism are some of the risk factors for OP [4, 5].

Furthermore, CVDs are one of the most common causes of death worldwide, and their prevalence has seen a steady increase [6]. Both OP and CVDs appear to share pathophysiological pathways and risk factors, such as aging and inflammation [7]. Several prospective studies have suggested that as women enter menopause, there is a marked increase in CVD risk, with a greater likelihood of myocardial infarction and all-cause mortality [8, 9]. Lampropoulos et al. have indicated that OP correlated with vascular calcification and hyperlipidemia [10]. Atherogenic dyslipidemia, that is, the combined occurrence of high fasting blood concentrations of triglycerides (TGs) and low levels of high-density lipoprotein cholesterol (HDL-C) is frequent among patients with metabolic disorders such as diabetes and metabolic syndrome [11, 12]. Comprehensive lipid ratios are considered to be better predictors for coronary artery disease than single lipid parameters [13]. In this sense, the atherogenic index of plasma (AIP) is defined as the base 10 logarithm of the ratio of the molar concentration of TG to HDL-C [13]. It has shown a good correlation with smaller LDL-C particles and with an increased fractional esterification rate for cholesterol in plasma, and it also represents a strong and independent predictor factor for coronary disease [14].

A high AIP has been used as a standalone index for estimating cardiac risk [15]. In addition, AIP was a strong predictor of atherosclerosis and myocardial infarction [16, 17]. Previous studies revealed that postmenopausal women were at an increased risk for developing CVD because of the elevated atherogenic lipid profile [18]. Conversely, several studies revealed that HDL-C and increased body weight are the protective factors of OP [19, 20].

In addition, studies focusing on OP prevalence and fragility fracture incidences are mainly concentrated in North and South Africa. National OP treatment guidelines have been published in Tunisia, Libya, Egypt, Morocco, and South Africa [21]. A recent study investigating the prevalence of hip fractures among black South Africans showed an age-adjusted hip fracture rate of 69.2 per 100,000 per annum and 73.1 per 100,000 per annum for women and men, respectively [22]. However, none of the sub-Saharan African countries, excluding South Africa, has a national guideline on the management of OP. Moreover, epidemiological studies reporting on the prevalence of hip fractures or vertebral fractures are limited in sub-Saharan Africa. The previous published study from sub-Saharan Africa was based on retrospective data, including small sample size, and from a single-study site [23]. Moreover, there is a scarcity of publications focusing on CVD risk among osteoporotic African women. In addition, the interplay between lipid and bone metabolism is an active field of research, due to the growing evidence for a biological linkage between two of the leading public health problems, that is, osteoporosis and atherosclerosis [24].

Sudan, one of the sub-Saharan African countries, suffers from the double burden of over and undernutrition. Recently, there have been reports of an increased prevalence of chronic diseases, including CVD. However, osteoporosis remains a neglected disease in Sudan, and there are no age-standardized reference data available to accurately screen and diagnose individuals suffering from such diseases. Furthermore, CVD risk factors among osteoporotic postmenopausal women have not been examined. Therefore, in this current study, we aimed to assess osteoporotic postmenopausal women's lipid profile, AIP, anthropometric measurements, and the possible risk for CVD. It is hypothesized that women would have elevated lipid profiles and high AIP and are obese, which increased their CVD risk.

## 2. Methods

### 2.1. Study Design and Participants

This is a cross-sectional, community-based study conducted between January 2018 and December 2019 in Khartoum state, the capital of Sudan. Various orthopedic outpatient clinics were visited to select the study sample, using a convenience sampling method. The inclusion criteria were as follows: postmenopausal women who reported having no menses at least for 1 year and aged ≥45 years. In addition, this study excluded women who smoked or consumed alcohol; those with a previous history of early or premature menopause; those with hepatic, renal, or thyroid dysfunction; those with heart disease, diabetes, and hypertension; those with hormonal replacement therapy, lipid-lowering drugs, and/or medications that could affect bone metabolism, lipid profile, or CVD risk.

### 2.2. Sample Size Calculation

At the time of the study, there was no published data on the prevalence of OP in Sudan. Thus, the prevalence of OP in Egypt (28.4%) [25], which is one of the neighboring countries, was used. The sample size was determined based on the following equation:

(1) $$\begin{array}{c} n=\frac{{\left(Z_{1-\alpha /2}\right)}^{2}\times P\left(1-P\right)}{d^{2}}, \end{array}$$

where *n* = sample size; *Z*_1−*α*/2_ = 1.465 for the 90% confidence level; *d* = desired margin of an error, expressed as a decimal (0.05); *P* = prevalence of the disease (28.4%); *n* = 222.

### 2.3. Diagnostic Criteria for Osteoporosis and Osteopenia

Dual-energy X-ray absorptiometry was used to measure BMD in g/cm^2^ in the proximal femur and lumbar spine using Hologic Inc. ASY00409 X-Ray Controller for Hologic Discovery Bone Densitometry. According to the symptoms, signs, and BMD (T-score), women were diagnosed and assessed for eligibility by an orthopedic doctor who used the World Health Organization (WHO) diagnostic classification [26]. Osteopenia was diagnosed with a *T*-score between −1 and −2.5, whereas osteoporosis with a *T*-score of −2.5 or lower. In this current study, we presented the results as women with a normal *T*-score, osteopenia, or osteoporosis.

## 3. Data Collection

### 3.1. Anthropometric Measurements

Weight was measured twice by following the published protocols. The OMRON body fat scale (BF508l, China) was used after being calibrated. Women were asked to remove heavy clothes, shoes, and accessories, and readings were taken to the nearest 0.1 kg. A portable stadiometer (SECA-213 model, Germany) was used after calibration to measure height twice, and women were asked to remove their shoes. The Quetelet body mass index (BMI) was calculated using the standard formula (weight in kilogram/height in meter square). The WHO classification was used to define the BMI as follows: underweight (<18.5 kg/m^2^), normal (18.5–24.9 kg/m^2^), overweight (25.0–29.9 kg/m^2^), and obese (≥30 kg/m^2^) [22].

Waist circumference (WC) was measured twice using a nonstretchable measuring tape. Women fasted for 8 hours. Measurements were made at the approximate midpoint between the lower margin of the last palpable rib and the top of the iliac crest. Readings were taken from the right side of the body. The cutoff values associated with increased CVD risks in women were set at >88 cm [27].

### 3.2. Blood Sampling and Biochemical Measurements

After a minimum of 8 hours of overnight fasting, peripheral blood (5 mL) was drawn in plain containers. Total cholesterol (TC), triglyceride (TG), low-density lipoprotein cholesterol (LDL-C), and high-density lipoprotein cholesterol (HDL-C) were assayed in serum samples on the Cobas c311 system (Roche Diagnostics GmbH, Germany). The precision and accuracy of the techniques used in this study were checked each time, and each batch was analyzed by including commercially prepared control sera. Serum TC was classified as follows: normal <200 mg/dL, borderline 200–239 mg/dL, and high ≥240 mg/dL; TG: normal <150 mg/dL, borderline high 150–199 mg/dL, and high ≥200 mg/dL; LDL-C: normal level <130, borderline high 130–159 mg/dL, high 160–189 mg/dL, and very high >189 mg/dL; HDL-C: optimal ≥60 mg/dL, borderline low 59–40 mg/dL, and low <40 mg/dL [28]. AIP was calculated as the logarithmical transformed ratio of molar concentrations of TG to HDL-C [25]. AIP was classified as follows: low CVD risk <0.1, medium risk 0.1–0.24, and high risk >0.24 [17].

Furthermore, the number of CVD risk factors among women was independently calculated based on their BMI (>25 kg/m^2^), WC (>88 cm), serum TC (>200 mg/dL), LDL-C (>130 mg/dL), and AIP (>0.1).

### 3.3. Quality Assurance

Data collectors attended three training sessions. These sessions included how to take anthropometric measurements, collect blood samples, and analyze blood samples.

### 3.4. Ethical Consideration

Ethical approval was obtained from the Deanship of Scientific Research at the Sudan University of Science and Technology (NO : DSR-IEC-03-08). All the procedures used in this study met the current revision of the Helsinki Declaration. The study protocol was explained to women who provided signed consent before the start of the study. It is important to note that the enrollment in the study was voluntary, and no incentives were provided to women.

### 3.5. Statistical Analysis

Data were coded, and statistical analysis was performed using the Statistical Package for the Social Sciences version 26.0 (SPSS Inc., Chicago, IL, USA). The Kolmogorov–Smirnov test was used for testing the normality of continuous data (age, WC, BMI, TC, TG, LDL-C, and HDL-C levels). Continuous variables with a normal distribution (BMI, WC, and AIP) were presented as the mean (SD), whereas continuous variables with a skewed distribution (age, TC, TG, LDL-C, and HDL-C) were presented as the median with interquartile ranges (IQ). The analysis of variance (ANOVA) test with post hoc multiple comparisons was used to determine the differences between the three groups of women. The median of the skewed data was compared using the Kruskal–Wallis test. Spearman's correlation was used to find the association between variables. The multiple linear regression model was also used to determine the most significant risk factors which affected the *T*-score values. The significance level was set at ≤0.05.

## 4. Results

Women (*n* = 400) were recruited, with 300 fulfilling the inclusion criteria, giving a response rate of 75%. Their age ranged between 47 and 90 years, and their postmenopausal peroid ranged between 2 to 45 years. As per our findings, women (*n* = 80, 26.6%) had normal *T*-scores, whereas 220 had either osteopenia (*n* = 110, 36.7%) or osteoporosis (*n* = 110, 36.7%) (Table 1). Women with normal *T*-scores were significantly younger than their osteopenic and osteoporotic counterparts (*P* < 0.001). Moreover, women with normal *T*-scores suffered from general and central obesity, whereas osteopenic and osteoporotic women were overweight (Table 1).

Furthermore, one-way ANOVA with post hoc multiple comparison analysis showed that women with normal *T*-scores had significantly higher BMI than osteopenic (*P*=0.000; 95% CI: 1.72–6.27) and osteoporotic (*P*=0.001; 95% CI: 1.31–5.79) women. However, no significant differences were determined between osteopenic and osteoporotic (*P*=0.613; 95% CI: −2.19–1.30) women. In addition, one-way ANOVA with post hoc multiple comparison analysis showed that women with normal *T*-scores had significantly higher WC than osteopenic (*P*=0.007; 95% CI: 1.62–12.67) and osteoporotic (*P*=0.021; 95% CI: 0.74–11.60) women. There were no significant differences between osteopenic and osteoporotic women (*P*=0.894; 95% CI: −6.04–4.10).

**Figure 1:**
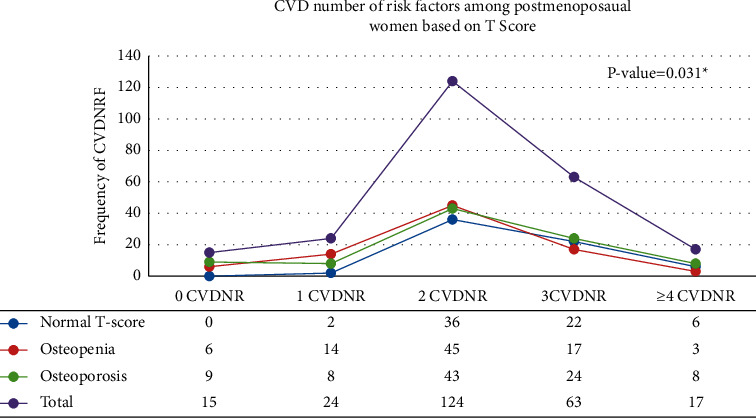
Cardiovascular disease number of risk factors (CVDNRF) among normal *T*-score, osteopenic, and osteoporotic postmenopausal women. ^*∗*^*P* value was obtained using the chi-square test. CVD risk factors included are as follows: body weight >25 kg/m^2^, waist ci.

Moreover, there were no significant differences among women with regard to their lipid profiles. Overall, 24.4% of the postmenopausal women had hypercholesterolemia (≥200 mg/dL), 25.6% had hypertriglyceridemia (≥150 mg/dL), and 13.7% had high levels of LDL-C (≥130 mg/dL) (Table 2). In addition, the HDL-C levels among most women (76%) were within the lower ranges. Based on their AIP values, 33.3% of women had medium to high CVD risk, although their mean AIP was within the normal range (Table 2). Furthermore, 61% of the postmenopausal women had one to two CVD risk factors (Figure 1). Many women with normal *T*-scores had ≥3 CVD risk factors.

AIP was positively associated with TC among osteopenic (*r* = 0.292, *P*=0.002) and osteoporotic women (*r* = 0.265, *P*=0.005). Among osteopenic women, AIP correlated positively with LDL-C (*r* = 0.21, *P*=0.025). Moreover, there was a negative relationship between HDL-C and age (*r* = −0.21, *P*=0.032) among osteoporotic women. In addition, AIP was found to have correlated positively with the number of CVD risk factors among women with normal *T*-scores (*r* = 0.522, *P* = 0.000) and osteoporotic (*r* = 0.418, *P*=0.000) women. The multiple linear regression model determined the factors affecting *T*-score values. The dependent variable entered in the model was *T*-score values, whereas age, BMI, TC, LDL-C categories, and AIP were independent variables. Findings revealed that *T*-score values decreased significantly with age and increased with the BMI (Table 3).

## 5. Discussion

Osteoporosis is known to be a metabolic disease that mainly affects older adults and is a major cause of morbidity and mortality [7]. However, studies examining osteoporosis and its prevalence remain limited, especially among women [23]. The disease is not yet considered a health priority in many low-middle-income countries, including sub-Saharan countries such as Sudan. Our study revealed that the prevalence of OP among postmenopausal Sudanese women was 36.7%, which is higher than the Turkish (16.2%) [29] and Egyptian (28.4%) [25]. On the contrary, the prevalence was lower than in other African countries such as Nigeria (65.8%) [30] and Cameroon (55.8%) [31]. The higher prevalence in African countries could be related to the differences in the study design, diagnostic techniques, the bone scan site chosen, and the selection of patients.

Furthermore, in this study, we revealed that women with normal *T*-scores suffered from general obesity (BMI of 33 kgm^−2^), whereas osteopenic and osteoporotic women were overweight. In addition, irrespective of the *T*-score, all women suffered from central obesity. Similar findings were reported among other populations including Chinese, Caucasian-Americans [32], and osteoporotic Nigerian women [33]. Larger body weight was associated with higher bone mass [19]. One explanation for this association could be due to the mechanism that relates the increased BMI to higher estrogen production and osteoclast suppression [34, 35]. In addition, estrogen is known to help in maintaining skeletal homeostasis; it also inhibits bone resorption, hence protecting bone [36]. Furthermore, obesity could help sustain BMD by causing a relative increase in bone formation, mineral density, and an increase in the number of adipocytes [37–39]. Thus, general obesity has protected some Sudanese women (*n* = 80) from developing OP.

Although obesity is protective against OP, both obesity (general and central) and overweight are major risk factors for CVD [40]. Thus, our hypothesis stating that women could be obese and at increased risk for CVD is accepted. Our findings agreed with those of other studies, e.g., study by Uyma et al. carried out among Cameroonian women [41]. These findings increased concern about menopausal transition, obesity, and CVD risk. Irrespective of having normal or low bone mineral densities, postmenopausal Sudanese obese women had increased CVD risk.

In this study, we hypothesized that women would suffer from elevated lipid profiles and AIP. We highlighted that dyslipidemia was prevalent among postmenopausal women. Thus, our study hypothesis is accepted. Moreover, there were no significant differences among the study groups regarding their lipid profile and AIP. Sudanese women had lower mean serum TC, LDL-C, TG, and HDL-C levels than their Iranian counterparts [42]. Moreover, the mean AIP values were lower among Sudanese women than Cameroonian women [41]. Contradictory to our findings, hyperlipidemia was associated with a low BMD among postmenopausal Japanese [43] and Iranian [44] women. Moreover, our study disagreed with the findings of the study by Kan et al. who reported that total serum cholesterol was inversely associated with BMD among osteoporotic women [45]. However, our results agreed with the findings stated by others [46–48], who found no significant association between the lipid profile and BMD among postmenopausal women. One possible explanation for dyslipidemia could be obesity among women, in particular central obesity.

A strong association between dyslipidemia and CVD risk had been previously reported. AIP is considered a standalone index for cardiac risk estimation [15]. Our work revealed a significant association between AIP, LDL-C, and TC among women with lower *T*-scores. Similar findings were also reported previously [49]. AIP correlated positively with the number of CVD risk factors among the normal *T*-score and osteoporotic Sudanese women. Our findings were in agreement with those of other studies [49].

Our study has some limitations that need to be considered. Thus, a longitudinal study should be conducted in the future to follow up on these postmenopausal osteoporotic women for a certain period and report the actual incidence of CVD.

Our findings are considered the first published study among postmenopausal osteoporotic Sudanese women who were assessed for CVD risk. It focused on dyslipidemia, AIP, and obesity. Our study could serve as a baseline for subsequent studies.

## 6. Conclusion

The current study raised concern about CVD risk among osteoporotic postmenopausal Sudanese women. More studies need to focus on women aged >45 years because our findings showed a high prevalence of osteoporosis, dyslipidemia, AIP, and obesity, and all were considered risk factors for CVD. In addition, necessary steps are required for public education by a multidisciplinary team and broader dissemination of information about osteoporosis, CVD risk, and their prevention.

## Figures and Tables

**Table 1:** Age and anthropometric measurements of normal *T*-score, osteopenic, and osteoporotic Sudanese women.

	Normal *T*-score	Osteopenia	Osteoporosis	Total	*P* value^*∗*^
Age (years)	*n* = 80, 26.7%	*n* = 110, 36.7%	*n* = 110, 36.7%	*n* = 300	
Mean (SD)^#^	56.8 (8.0)	60.3 (8.9)	66.4 (10.9)	61.6 (10.2)	
Median (SEM)^†^	55.0 (0.9)	60.0 (0.8)	65.5 (1.0)	61.0 (0.5)	0.0001^§^
Q1–Q3	50.0–63.0	52.7–65.0	58.7–75.0	52.0–69.0	

Age groups (years)					
45.0–64.0	65.0 (81.3)^‡^	70.0 (63.6)	43.0 (39.1)	178.0 (59.3)	0.0001^a^
65.0–90.0	15.0 (18.8)	40.0 (36.4)	67.0 (60.9)	122.0 (40.7)	

BMI (kg/m^2^)	*n* = 66	*n* = 85	*n* = 92	*n* = 243	
Mean (SD)^#^	33.1 (6.1)	29.1 (5.8)	29.5 (5.7)	30.3 (6.1)	0.0001^*∗*^

BMI classification					
Normal (18.5–24.9 kg/m^2^)	5.0 (7.6)^‡^	21.0 (24.7)	23.0 (25.0)	49.0 (20.2)	0.003^a^
Overweight (25–29.9 kg/m^2^)	14.0 (21.2)	27.0 (31.8)	16.0 (17.4)	57.0 (23.5)	
Obese (≥30 kg/m^2^)	47.0 (71.2)	37.0 (43.5)	53.0 (57.6)	137.0 (56.4)	

WC (cm)	*n* = 66	*n* = 85	*n* = 92	*n* = 243	
Mean (SD)^#^	108.6 (13.8)	101.5 (11.5)	102.5 (16.6)	103.8 (14.5)	0.006^*∗*^

WC classification					
≤88.0 cm	4.0 (6.1) ^‡^	13.0 (15.3)	19.0 (20.7)	36.0 (14.8)	0.039^a^
>88.0 cm	62 (93.9)	72.0 (84.7)	73.0 (79.3)	207.0 (85.2)	

^#^Mean, geometric mean; SD, standard deviation; ^†^S.E.M, standard error of mean, ^‡^values are numbers and percentages; ^§^*P* value is obtained using the Kruskal–Wallis test, ^*∗*^, *P* value is obtained using ANOVA with post hoc multiple comparison analysis, ^a^*P* value is obtained by the chi-square test.

**Table 2:** Lipid profile of normal *T*-score, osteopenic, and osteoporotic postmenopausal Sudanese women.

	Normal *T*-score *n* = 80, 26.6%	Osteopenia *n* = 110, 37.7%	Osteoporosis *n* = 110, 37.7%	Total *n* = 300	*P* value^*∗*^
Total cholesterol (mg/dL)					
Mean (SD)^#^	173.6 (38.1)	174.6 (44.3)	181.9 (41.3)	177.0 (41.6)	
Median (S.E.M)	176.0 (4.2)	170.5 (4.2)	182.5 (3.9)	177.0 (2.4)	0.124^*∗*^
Q1–Q3	146.7–197.5	141.0–194.2	159.7–200.0	149.2–199.0	

TC classification					
Normal <200.0	61.0 (76.3)^‡^	85.0 (77.3)	81.0 (73.6)	227.0 (75.7)	0.712^a^
Borderline 200.0–239.0	16.0 (20.0)	17.0 (15.5)	20.0 (18.2)	53.0 (17.7)	
High ≥240.0	3.0 (3.8)	8.0 (7.3)	9.0 (8.2)	20.0 (6.7)	

Triglycerides (mg/dL)					
Mean (SD)^#^	125.7 (39.7)	120.0 (51.4)	123.3 (49.8)	122.7 (47.9)	
Median (SEM) ^†^	119.0 (4.4)	109.0 (4.9)	108.5 (4.7)	111.0(2.7)	0.293^*∗*^

Q1–Q3	101.2–150.7	89.0–141.7	86.5–154.2	90.0–150.0	

TG classification					
Normal <150.0	59.0 (73.8)^‡^	88.0 (80.0)	76.0 (69.1)	223.0 (74.3)	0.307^a^
Borderline high 150.0–199.0	16.0 (20.0)	15.0 (13.6)	21.0 (19.1)	52.0 (17.3)	
≥200.0	5.0 (6.3)	7.0 (6.4)	13.0 (11.8)	25.0 (8.3)	

LDL-C (mg/dL)					
Mean (SD)^#^ mg/dL	93.9 (27.0)	92.5 (32.8)	99.5 (32.1)	95.4 (31.1)	
Median (SEM) ^†^	90.5 (3.0)	88.3 (3.1)	99.0 (3.0)	93.0 (1.8)	0.169^*∗*^
Q1–Q3	75.7–112.7	71.0–110.0	74.7–118.5	74.0–115.0	

LDL-C classification					
Normal <130.0	70.0 (88.6)^‡^	96.0 (87.3)	92.0 (83.6)	258.0 (86.3)	0.596^a^
Borderline high 130.0–159.0	8.0 (10.1)	9.0 (8.2)	14.0 (12.7)	31.0 (10.4)	
High 160.0–189.0	1.0 (1.3)	5.0 (4.5)	4.0 (3.6)	10.0 (3.3)	

HDL-C (mg/dL)					
Mean (SD)^#^ mg/dL	50.2 (15.5)	47.8 (15.7)	48.9 (15.0)	48.8 (15.3)	
Median (S.E.M)^†^	47.0 (1.7)	45.5 (1.4)	47.0 (1.4)	46.5 (0.8)	0.470^*∗*^
Q1–Q3	39.0–61.0	35.0–57.5	38.0–58.0	37.0–58.0	

HDL-C classification					
Low ≤40.0	24.0 (30.0)	44.0 (40.0)	36.0 (32.7)	104.0 (34.7)	0.510^a^
Borderline low 59.0–41.0	34.0 (42.5)	40.0 (36.4)	50.0 (45.5)	124.0 (41.3)	
Optimal ≥60.0	22.0 (27.5)^‡^	26.0 (23.6)	24.0 (21.8)	72.0 (24.0)	

Atherogenic index of plasma					
Mean (SD)	0.037 (0.1)	0.033 (0.1)	0.026 (0.2)	0.032 (0.1)	0.928^†^
Median (S.E.M)^†^	0.040 (0.01)	0.010 (0.01)	−0.002 (0.02)	0.017 (0.01)	
Q1–Q3	−0.087–0.135	−0.094–0.139	−0.130–0.159	−0.111–0.142	

AIP classification					
Low risk <0.1	52.0 (65.0)^‡^	78.0 (70.9)	70.0 (63.6)	200.0 (66.7)	0.137^a^
Medium risk 0.1–0.24	20.0 (25.0)	13.0 (11.8)	22.0 (20.0)	55.0 (18.3)	
High risk >0.24	8.0 (10.0)	19.0 (17.3)	18.0 (16.4)	45.0 (15.0)	

^#^Mean, geometric mean; SD, standard deviation; ^†^median; S.E.M, standard error of mean; Q1–Q3 interquartile ranges; ^‡^values are numbers and percentages; ^†^*P* value was obtained using ANOVA test with post hoc multiple comparison analysis to compare between means; ^*∗*^*P* value was obtained using the Kruskal–Wallis test to compare between medians; ^a^*P* value was obtained using the chi-square test to compare between frequencies.

**Table 3:** Multiple linear regression with the diagnosis^^*∗*^^ being the dependent variable.

	Unstandardized coefficients		Standardized coefficients	*T*	Sig.	95% CI^#^	
	*B*	Std. error	Beta			Lower bound	Upper bound
Age	0.028	0.005	0.358	6.031	0.000	0.019	0.037
BMI	−0.024	0.008	−0.183	−3.076	0.002	−0.039	−0.009
TC	0.002	0.001	0.091	1.299	0.195	−0.001	0.004
LDL-C Categories	−0.017	0.124	−0.009	−0.135	0.893	−0.262	0.228
AIP	−0.179	0.262	−0.041	−0.686	0.493	−0.695	0.336

^*∗*^Diagnosis=refer to having normal *T*-score, osteopenia, or osteoporosis; ^#^CI=confidence interval. Age, BMI, TC, LDL-C categories, and AIP were the independent variables entered in the regression model. The sum of squares = 153.98, *R*^2^ = 0.184, *R* = 0.428, adjusted *R*^2^ = 0.166, df2 = 236, F change = 10.611, and significance = 0.000.

## Data Availability

The datasets used and/or analyzed during the current study are available at the link https://figshare.com/articles/dataset/Postmenopausal_women_BMD/19768294.
